# Polygenic Risk Score Improves Cataract Prediction in East Asian Population

**DOI:** 10.3390/biomedicines10081920

**Published:** 2022-08-08

**Authors:** Chih-Chien Hsu, Hao-Kai Chuang, Yu-Jer Hsiao, Yuan-Chi Teng, Pin-Hsuan Chiang, Yu-Jun Wang, Ting-Yi Lin, Ping-Hsing Tsai, Chang-Chi Weng, Tai-Chi Lin, De-Kuang Hwang, Ai-Ru Hsieh

**Affiliations:** 1 School of Medicine, National Yang Ming Chiao Tung University, Taipei 112304, Taiwan; 2 Department of Ophthalmology, Taipei Veterans General Hospital, Taipei 112027, Taiwan; 3 Department of Medical Research, Taipei Veterans General Hospital, Taipei 112027, Taiwan; 4 Department of Statistics, Tamkang University, New Taipei 251301, Taiwan

**Keywords:** cataract, genome-wide association studies, polygenic risk score, Asian population, biobank, retrospective study

## Abstract

Cataracts, characterized by crystalline lens opacities in human eyes, is the leading cause of blindness globally. Due to its multifactorial complexity, the molecular mechanisms remain poorly understood. Larger cohorts of genome-wide association studies (GWAS) are needed to investigate cataracts’ genetic basis. In this study, a GWAS was performed on the largest Han population to date, analyzing a total of 7079 patients and 13,256 controls from the Taiwan Biobank (TWB) 2.0 cohort. Two cataract-associated SNPs with an adjustment of *p* < 1 × 10^−7^ in the older groups and nine SNPs with an adjustment of *p* < 1 × 10^−6^ in the younger group were identified. Except for the reported AGMO in animal models, most variations, including rs74774546 in GJA1 and rs237885 in OXTR, were not identified before this study. Furthermore, a polygenic risk score (PRS) was created for the young and old populations to identify high-risk cataract individuals, with areas under the receiver operating curve (AUROCs) of 0.829 and 0.785, respectively, after covariate adjustments. Younger individuals had 17.45 times the risk while older people had 10.97 times the risk when comparing individuals in the highest and lowest PRS quantiles. Validation analysis on an independent TWB1.0 cohort revealed AUROCs of 0.744 and 0.659.

## 1. Introduction

Cataracts are the leading cause of blindness globally, with the proportion of cataract-induced blindness ranging from 12.7% in North America to 42% in Southeast Asia [1]. It is an eye disease characterized by the opacification of the crystalline lens, a biconvex structure in human eyes that converges light and focuses images on the retina. Opacification in crystalline lenses causes blurry or scattered vision and glare problems, eventually leading to vision loss. The diagnosis of cataracts involves confirming the lens opacity through a slit lamp examination. Once the severity of cataracts interferes with patients’ demands of daily living, intervention is needed. However, the current therapeutic medications based on quinoid and free radical theories of the development of cataracts have shown little efficacy [2,3]. The only effective treatment relies on surgery aimed at replacing the opaque, cataractous lens with a transparent artificial one. This approach, however, has its limitations and complications. For example, a monofocal intraocular lens cannot adjust the focus, while a multifocal intraocular lens has the disadvantages of glare, light scattering, and halo at night. When complications such as endophthalmitis or suprachoroidal hemorrhage occur, the patient’s vision may be severely impaired even after surgery. In the post-genomic era, investigation into cataract-related genes that allows for risk stratification by genetic variations opens new avenues into medical treatment and early intervention for cataracts.

The most common form of cataract is age-dependent among the various types of cataracts, such as childhood, secondary to trauma, glaucoma, infection-related, and so on [4]. It is influenced by a range of environmental and genetic factors [5,6], including age, sunlight exposure [7], smoking [8], alcohol, metabolic syndrome [9], and iatrogenic corticosteroids [10]. Twin studies and family aggregation studies, on the other hand, have revealed that genetic factors play a role in the development of senile cataracts, with the estimated heritability ranging from 21% to 64% [11]. In a multi-ethnic genome-wide association study (GWAS), 54 cataract risk loci with several potential drug targets, such as RARB, KLF10, DNMBP, HMGA2, MVK, BMP4, CPAMD8, and JAG1, were identified [12]. Most GWAS analyses of cataracts have been limited to only the Western population. In 2014, the first large-scale meta-analysis in multiethnic Asians identified two loci for age-related nuclear cataracts, namely rs7615568 on the KCNAB1 gene, and rs11911275 on the CRYAA gene [13]. However, heterogeneities among the associations were found due to the recruitment of the multiethnic Malay and Indian individuals in the study.

Here, we present the largest pure Han population GWAS on cataracts in the Taiwan Biobank (TWB) 1.0 and 2.0 databases, with more than 144,000 Taiwanese individuals from both the community and teaching hospitals (http://www.twbiobank.org.tw/, accessed on 25 February 2022) [14]. Genome-wide SNP data were collected from custom SNP arrays versions 1.0 and 2.0. Principal components analysis of the genetic data confirmed that over 99% of the TWB participants are Han Chinese, differing from previous multiethnic GWAS research on cataracts [14,15]. In this study, 11,110,260 SNP variants from 20,335 TWB2.0 participants were analyzed. Cataract-related SNP loci were identified by GWAS, and differential genetic architecture for young cataract patients (aged under 60) and old cataract patients (aged over 60) is illustrated in Manhattan plots. Finally, we constructed a polygenic risk score (PRS) to identify high-risk cataract individuals, and the results were validated in 5-fold cross-validation subsets. Replication was performed on TWB1.0. We further compared our association results with BioBank Japan (http://jenger.riken.jp/en/result, accessed on 25 February 2022) and the UK Biobank (https://pheweb.org/UKB-TOPMed, accessed on 25 February 2022) (Tables S1–S3).

## 2. Materials and Methods

### 2.1. Study Population and Genome-Wide Association Study

The participants and their data were obtained exclusively from the TWB (https://www.biobank.org.tw/, accessed on 25 February 2022). The Taiwan Biobank (TWB) collects extensive phenotypes, including demographics, socioeconomic status, environmental exposures, lifestyle, dietary habits, family history, and self-reported disease status, through structured questionnaires. Up to 15 April 2021, more than 144,000 participants were recruited. The demographic and health-related survey data for 105,388 study subjects were released in December 2019. Detailed genotyping and the imputation procedure are described by Wei et al. [14]. In brief, 105,388 demographic and health-related survey data were released in December 2019. There were 95,252 participants who had been genotyped with custom TWB1.0 array (TWB1.0 = 27,737) or TWB2.0 array (TWB2.0 = 68,978).

The control samples in this study were restricted to individuals aged 60 or over because most cataracts are age-related. Sample quality control was carried out to exclude samples and SNP markers based on the following criteria: (i) with a missing call rate > 2%, (ii) with a minor allele frequency (MAF) < 1%, or (iii) with significantly deviated from the Hardy-Weinberg equilibrium (*p* < 1.0 × 10^−6^) using PLINK (v1.9) [16].

After performing quality control for the samples, 11,785,052 variants from 7993 (3003 cases, 4990 controls) TWB1.0 and 11,110,260 variants from 20,335 (7079 cases, 13,256 controls) TWB2.0 participants were used in the subsequent analysis. After sex, age, diabetes, hypertension, dyslipidemia, asthma, glomerular filtration rate, and body mass index were adjusted as covariates, logistic regression analysis was performed using PLINK software.

### 2.2. Polygenic Risk Score (PRS) Analyses

To build the PRS prediction models, we used the standard clumping and thresholding (C + T) method. The hyperparameters for this method were the cut-off of correlation r^2^ and *p*-value threshold *p*. The parameter spaces for r^2^ and *p* were {0.2, 0.04} and {10^−4^, 2.5 × 10^−4^, 5 × 10^−4^, 7.5 × 10^−4^, 10^−5^}, respectively. For each combination of (r^2^, *p*), we used PLINK with a window size of 10 Mb to select SNPs. For the model selection, we considered TWB2.0 as the training sample to report the prediction performance (AUC) and TWB1.0 as the testing sample to evaluate the AUC of the prediction model. For the SNPs whose minor alleles showed protective effects on cataracts, we converted their minor alleles to major alleles as risk alleles, which resulted in positive weight values for all variants. For the genetic risk estimation, individuals were divided into quintiles based on the PRS values in each study cohort. The (min,Q1) group was defined as the minimum and bottom 25% of the PRS values, the (Q1,Q2) group was defined as the bottom 25% and bottom 50% of the PRS values, and the same applies to the other groups. We calculated the PRS as the weighted sum of the risk alleles $\sum_{i=1}^{k}\beta_{i}SNP_{i}$, where k is the number of SNPs, $SNP_{i}$ is the number of risk alleles, and $\beta_{i}$ is the coefficient of logistic regression [17]. The PRS analyses were performed using PLINK 1.9. In this study, the predictive abilities of TWB1.0 and TWB2.0 PRS were compared using the area under the receiver operating characteristic curve (AUROC) [18]. The analyses were performed using the R package “pROC”.

### 2.3. Cross-Validation

Cross-validation (CV) is a model training method that can assess prediction accuracy [19]. Since TWB is not split into training or testing data, we resorted to five-fold CV, which is often used in machine-learning modeling [20,21]. Five-fold CV is used to assess how well a classification model generalizes to independent datasets and splits the dataset into five equal and mutually exclusive subsets. Then, each of the subsets is used once for testing (with the other four being used for training). This process is repeated five times, with each of the five subsets being tested only once.

## 3. Results

### 3.1. Participant Characteristics in TWB 2.0 and TWB 1.0

A total of 68,978 TWB2.0 participants and 27,737 TWB1.0 participants were analyzed in our study, with the TWB2.0 participants as the cataract risk loci discovery set and those of TWB1.0 as the validation set. Furthermore, 10.2% (7079/68,978) of the individuals in TWB2.0 who passed our quality control could be set as the discovery set (Table 1). Among the 7079 individuals, 1959 patients were under 60 years old, while 5120 patients were older than 60 years old. In subsequent discussions, the former group will be referred to as the younger group, while the latter as the older group. The 13,256 individuals fitting our quality control from the remaining participants were considered the control group, making the total sample size for the discovery set 20,335. On the other hand, in the validation set (TWB1.0), there were 7993 individuals, consisting of 3003 self-reported cataract cases and 4990 controls. Among the cases, 757 individuals belonged to the younger group. The baseline characteristics for both the discovery and validation sets are shown in Table 1. In the discovery set, females predominated among the cases (71%) compared to the controls (63%). The mean ages of the older and younger cases were 65 and 54 years old, respectively. Since age, sex, diabetes, body mass index (BMI), hypertension, asthma, and chronic kidney diseases are statistically heterogeneous between the cases and controls, all of these were adjusted in the following analysis.

### 3.2. Cataract Risk Loci

**Figure 1:**
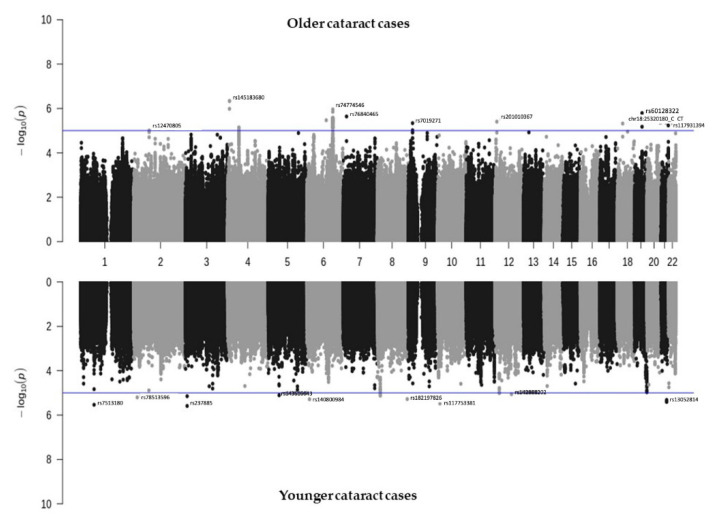
Manhattan plot showing the SNPs associated with cataracts identified from TWB2.0. Older cataract cases (≥60 years old, *n* = 5120) are shown in the top panel, while younger cataract cases (<60 years old, *n* = 1959) are shown in the bottom panel.

To investigate which SNPs are significantly associated with cataracts, we performed a GWAS on TWB2.0, and a mirror Manhattan plot was generated after the covariate adjustment (Figure 1, Tables S1–S3). A Bonferroni-corrected significance threshold of *p* = 4.5 × 10^−9^ (0.05/11,110,260) was prespecified to adjust for multiple testing. However, Bonferroni correction is thought to be too stringent and conservative [22]. Hence, associations with p-values between 1.01 × 10^−7^ and 1.01 × 10^−5^ were considered suggestive associations, and those between 1.01 × 10^−7^ and 4.5 × 10^−9^ were considered putative associations [23,24,25]. In the older group, 167 SNPs related to cataracts showed values of less than 1 × 10^−5^, including two SNPs with adjustments of <1 × 10^−7^, 142 with adjustments of *p* < 1 × 10^−6^, and 23 with adjustments of <1 × 10^−5^. In the younger cataract group, there were nine SNPs with adjustments of <1 × 10^−6^, and 31 SNPs with adjustments of <1 × 10^−5^. There were no overlapping SNPs with <1 × 10^−5^ in both the younger and older groups. The selected SNPs are listed in Table 2 and the details are listed in Tables S1–S3. While most SNPs were not replicated in other biobanks, rs9788929 in the gene XYLT1 was shown to be <0.05 in the UK Biobank, and rs2272537 in the gene ZBTB32, rs56792854 in KMT2B, and rs60128322 in PROSER3 were <0.05 in the BioBank Japan (Tables S1–S3).

### 3.3. Polygenic Risk Score (PRS) and Cataract Risk Prediction

**Figure 2:**
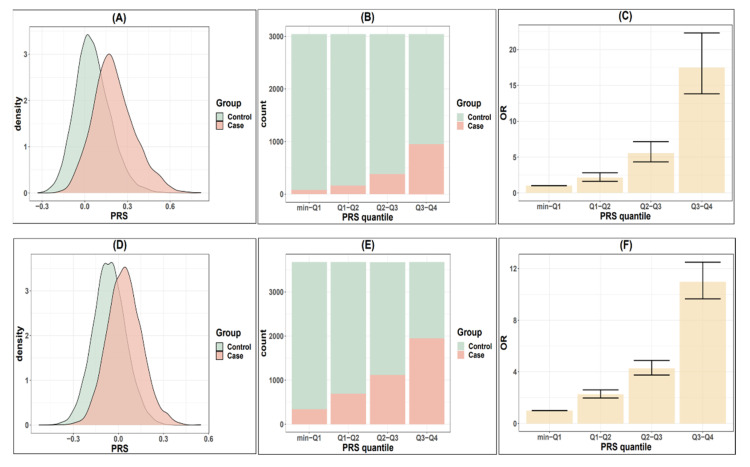
Comparison of cataract risks in TWB2.0 classified by PRS quantile. (**A**) Distribution of the polygenic risk score (PRS_younger) in younger cataract cases (<60) and controls. (**B**) Distribution of younger cases and controls according to PRS_younger quantiles. (**C**) Odds ratio for developing cataract in younger population according to PRS_younger quantiles. (**D**–**F**) are the cataract risks classified by PRS_older in older populations (≥60 years old).

To predict cataract risk, we constructed PRS models for both the younger and older cataract groups based on the associated SNPs discovered from the TWB2.0. Table 3 shows different models based on various combinations of the linkage disequilibrium (LD) clumping threshold (r^2^) and the genome-wide significance level threshold (*p*). The mean PRS was significantly higher among the cataract cases compared to the controls across all models in both groups (Table 3 and Figure 2A,D). Considering the clinical significance, r^2^, *p*, and AUC, we constructed the model with 218 selected independent SNPs for the younger cataract group (r^2^ < 0.04, and *p* < 2.5 × 10^−4^, referred to as PRS_younger), and 287 SNPs for the older cataract group (r^2^ < 0.04, and *p* < 2.5 × 10^−4^, referred to as PRS_older).

Regarding the PRS performance, the PRS_younger and PRS_older effectively distinguish individuals with high cataract risk from those with low risks in the younger and older groups, respectively (Figure 2A,D). Such an association demonstrates a dose-response effect (Figure 2B,C,E,F, Table 4). In the younger group, the individuals in the highest quantile of PRS_younger (Q3,Q4) demonstrated a 17.45-fold increase in risk compared to those in the lowest risk quantile (min,Q1). The second (Q2,Q3) and third (Q1,Q2) highest quantiles showed 5.52- and 2.12-fold increases in risk, respectively, compared to the lowest group. In the older group, the odds ratios were 10.97, 2.48, and 2.26 for the individuals in the highest, second highest, and third highest quantiles compared to those in the lowest quantile (min,Q1). Table 4 shows the case-control distribution among the quantiles. Furthermore, in the high-risk group (the top 5% to 25% in the PRS distribution), Table 5 shows a significantly elevated risk of cataracts compared to the remaining population. For the younger group, the top 25% of the PRS had a 6.24-fold increased risk, the top 10% had a 7.09-fold increased risk, and the top 5% had a 9.16-fold increased risk of developing cataracts compared to the remaining population. For the older group, the relative risk was 4.63, 5.48, and 6.74 when comparing the top 25%, 10%, and 5% groups to the remaining population.

**Figure 3:**
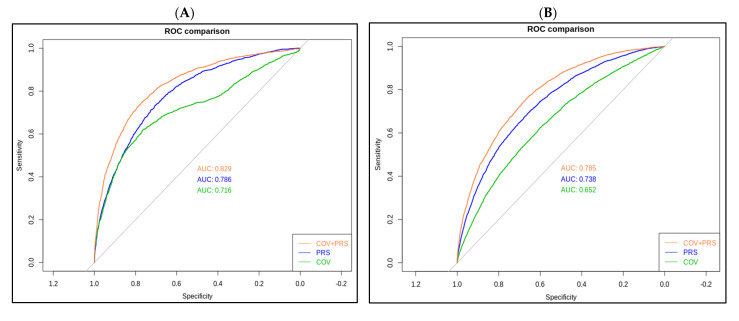
Receiver operating characteristic (ROC) curves for the polygenic risk score (PRS) model. (**A**) PRS_younger refers to younger cataract cases (<60), and (**B**) PRS_older refers to the older cataract cases (≥60).

In PRS_younger and PRS_older model, the area under the receiver operating curve (AUROC) were 0.786 and 0.738, respectively (Figure 3). After additional covariates were included in the model, the AUROC reached 0.829 and 0.785 in the younger and older groups, respectively (orange curves, Figure 3A,B). Replication results in TWB1.0 showed that AUROC was 0.744 in the younger group and 0.659 in the older group.

## 4. Discussion

In this study, we included 20,335 individuals (7079 cases and 13,256 controls) from the Taiwan Biobank to identify cataract risk loci and build a polygenic risk score (PRS). We used the genotype data, as well as extensive phenotypes, including demographics, socioeconomic status, environmental exposures, lifestyle, dietary habits, family history, and self-reported disease status, collected using structured questionnaires answered by a Taiwanese population who are mostly of Han Chinese ancestry. According to a recent study investigating the population admixture of the Han Chinese residing in Taiwan, the Taiwanese subpopulations demonstrate high genetic homogeneity given Taiwan’s population structure and migration history [15]. Han Chinese ancestry is a less studied population in cataract-related research and is necessary for solving the genetic puzzle of cataractogenesis.

The importance of clarifying the molecular mechanism of cataracts—the world’s leading cause of blindness [26]—cannot be overstated. Cataracts, which can be defined as an opacity of the crystalline lens, are produced by the misfolding and aggregation of proteins [1] that adversely affect the transmission of light on the retina. Because genetic mutations and environmental stress can affect the protein-folding process in different ways, the molecular mechanisms of how disruptions to the crystalline lens protein stability, solubility, and interactions [27,28,29] result in cataracts remain unclear. Lens proteins undergo various alterations, including oxidative, osmotic, and other stresses [30]. Meanwhile, the study of gene polymorphism and new molecular markers may reveal the stresses associated with cataracts.

A total of 209 cataract-associated SNPs at a significance level of *p* < 1 × 10^−5^ were identified in our GWAS. Most of the identified SNPs were unreported, including the topmost SNPs, rs74774546 in GJA1, rs237885 in OXTR, and others. While mapping a list of newly-identified loci from the GWAS to genes is a known challenge, it is nonetheless necessary and relevant for further functional follow-ups. In this work, the 209 cataract-associated SNPs were mapped to a list of 30 genes (Tables S1 and S2). HMX1, GJA1, and PROSER3 were identified to be the leading genes associated with cataracts in our older population. Additionally, there were seven SNPs intersecting the groups containing all the cataract cases and those aged above 60; three of which (rs76840465, rs28433905, and rs60128322) could be mapped to the AGMO, SCFD2, and PROSER3 genes. Furthermore, some SNPs identified in the younger population can be mapped to CAV3, OXTR, ROR1, and ERG genes. We also verified the SNPs identified in TWB2.0 in the UK Biobank (UKB) and BioBank Japan (BBJ). The gene XYLT1 was identified in our younger population and replicated in the UKB at a *p* < 0.05; the genes ZBTB32, KMT2B, and PROSER3 were identified in the older group and replicated in BBJ at a p-value < 0.05. Although all of these SNP associations with cataracts remain unclear and unreported, we must not rule out their relevance to the disease. We provide a brief description of some of their essential functions below that could help guide future follow-up experiments and pathway analyses into their mechanisms related to cataracts.

Firstly, the H6 family homeobox 1 (HMX1) gene is the leading SNP identified in our older population. It is located at 4p16.1. A previous genome-wide study of two individuals from a consanguineous family found an association of HMX1 with congenital cataracts. A homozygous missense mutation (c.650A>C; p.(Gln217Pro)) that abrogates the HMX1 function results in a rare oculoauricular syndrome associated with congenital cataracts, anterior segment dysgenesis, and retinal dystrophy [31,32]. Although rs145208055, located near HMX1, was identified in our older population, the linkage between senile cataracts and HMX1 remains to be explored.

The gap junction protein alpha 1 (GJA1) gene, located on chromosome 6 at the location of 6q22.31, was identified in the older population, and it encodes a connexin protein responsible for intercellular transmembrane channels at gap junctions. The channels provide a communication route for the diffusion of molecules between neighboring cells and play a particularly crucial role in the heart and embryonic development [33,34]. This gene is also related to the signaling receptor binding and protein domain-specific binding pathways. The GJA1 gene has been associated with oculodentodigital dysplasia [35], autosomal recessive craniometaphyseal dysplasia [36], and heart malformations [37], and may also play a role in the physiology of hearing by participating in the recycling of potassium to the cochlear endolymph and in cell growth inhibition [38]. GJA3 and GJA8, but not GJA1, have been associated with cataracts in previous studies. GJA3 and GJA8 are expressed on the specialized lens fibers that maintain the homeostasis and transparency of the lens [39]. However, the evidence for the involvement of the GJA1 gene in cataracts remains unclear.

The sec1 family domain (SCFD2) gene, identified in the older group, is a protein-coding gene that participates in protein transport and exocytosis [40] and is involved in multiple personality disorders. In terms of its potential role in ophthalmic diseases, a previous study has found the opposite effect of the Scfd2 gene on STAT1 and miR-493 regulators, which are associated with ischemia, a type of common pathological pathway for neuronal cell degeneration associated with many retinal diseases [41,42]. Other results from GWAS analysis also indicated that several variants within the SCFD2 gene locus achieved genome-wide statistical significance in their association with cataracts in the Australian Shepherd breed of domestic dogs [43]. Additionally, the SCFD2 gene has been associated with adiposity and diabetes [44].

The alkylglycerol monooxygenase gene (AGMO), identified in the older population, is a protein-coding gene located at 7p21.2. It is a tetrahydrobiopterin- and iron-dependent enzyme that cleaves the O-alkyl bond of ether lipids. The protective roles of AGMO against cataractogenesis, central nervous system myelination abnormalities, and spermatogenesis arrest have been proven based on the phenotypical report of ether lipid-deficient mice [45,46]. Additionally, AGMO may play a role in the development of type II diabetes [47], which is a risk factor for cataracts.

The proline- and serine-rich 3 (PROSER3) gene, identified in the older group, is located at the location 19q13.12. PROSER3 has shown its associations in previous GWAS with serum albumin [48], calcium [49], and sex hormone-binding globulin measurements [50]. Nonetheless, there have been no reported associations with cataracts and other eye diseases or molecular pathways so far.

The SNP rs237885, identified in the younger cataract group, is mapped to the oxytocin receptor (OXTR) gene located at 3p25.3. Such a gene locus also contains the coding region for caveolin 3 (CAV3). OXTR is a G-protein-coupled receptor that activates a phosphatidylinositol–calcium second messenger system [51]. The oxytocin–oxytocin receptor system plays a crucial role in the uterus during parturition and lactation and is associated with prosopagnosia [52]. In addition, the gene is related to pathways, including myometrial relaxation, contraction pathways, and RET signaling [53,54]. Additionally, OXTR is also found expressed in the amacrine cells of the inner nuclear layer. Transcriptome analysis revealed that the gene is implicated in the neuroactive ligand–receptor interaction, calcium signaling pathway, and cAMP signaling pathways during age-related transcriptional changes in the human retinal pigment epithelium (RPE) [55]. Although there are no existing studies on the roles oxytocin receptors play in the cataract molecular mechanism, epidemiological studies have found that breastfeeding is associated with a decreased likelihood of acquiring cataracts [56], with a largely unexplored molecular mechanism. It should also be noted that the oxytocin system has been associated with diabetes and adiposity [57].

The caveolin 3 (CAV3) gene located at 3p25.3 encodes caveolin proteins that are components of the caveolae plasma membranes. It interacts with and regulates G-proteins and voltage-gated potassium channels. The gene is involved in pathways, including smooth muscle contraction and the remodeling of adherens junctions. CAV3 mutations lead to disruptive protein oligomerization or intracellular routing, and further causes limb-girdle muscular dystrophy type-1C (LGMD-1C) [58], hyperCKemia [59], or rippling muscle disease (RMD) [60]. While the muscular manifestations in RMD are often misinterpreted as myotonia [61], and there is no known link between the two conditions, the association between CAV3 and myotonia presents with cataract symptoms and remains to be investigated.

ETS transcription factor ERG (ERG) is located on chromosome 21 at 21q22.2. Its association with cataracts was identified in the younger group in this study. This gene encodes a transcription factor belonging to the erythroblast transformation-specific (ETS) family, which comprises the key regulators of embryonic development, cell proliferation, differentiation, angiogenesis, inflammation, and apoptosis [62,63]. The protein is required for inducing vascular cell remodeling and regulating hematopoiesis. The translation of the ERG gene gives rise to different fusion gene products, such as TMPSSR2-ERG and NDRG1-ERG in prostate cancer, EWS-ERG in Ewing’s sarcoma, and FUS-ERG in acute myeloid leukemia [64,65]. Despite over two dozen recombination variants reported, the functions of these variants have not been determined, and the association with cataracts and other eye diseases remains to be unraveled.

In addition to finding the molecular pathway of cataracts, we also expanded this analysis to derive a polygenic risk score (PRS) that predicts cataract risks. The statistical significance of the PRS model supports the multifactorial nature of cataracts. Previous studies have created a PRS model containing six SNPs to predict cataract risks. Their results illustrated a 2.47-fold increase in risks in the high PRS group compared to the low PRS group after covariate adjustments [66]. Here, we provide the result of 200 independent SNPs. In our model, the younger patients within the highest quantile of PRS had a 17.45-fold increased risk of acquiring cataracts than those in the lowest quantile. Older patients in the highest PRS quantile had a 10.97-fold increased risk. Thus, our model offers a sufficient genetic tool to recognize high-risk cataract groups early. Additionally, since the number of older cases is three times more than that of younger cases, it is reasonable to confirm that genetics play a larger role in the younger population than in the older population. Additionally, the area under the curve can be improved by adding comorbidities such as aging and diabetes. This suggests that the interplay between environmental and genetic factors functions in the development of cataracts. Furthermore, this is the largest Taiwanese-based PRS cataract prediction model to date, proving its potential for clinical applications.

The advantage of the large-scale multi-center biobank in this study allowed us to determine cataract risk with great statistical power. Previous studies have presented the differential genetic landscape of cataracts among ethnic groups. In the comparison with BBJ and the UK Biobank, the results support the genetic disparity between the Han population and other ethnic groups and provide hints to common cataractogenic molecular pathways given the validation of the SNPs in BBJ and the UK Biobank. As for the PRS model, it exhibits potential for clinical application in the post-genomic era. Such a PRS model may aid ophthalmologists in prompting high-risk individuals to avoid modifiable risk factors, such as UV exposure or steroid usage. Early recognition and the prevention of cataracts may, as a result, reduce the demands of surgery. Nonetheless, our study is limited to the unavailability of data such as the age at diagnosis, clinical verification of diagnosis, and environmental risk factors, such as UV exposure or steroid usage. In addition, the inability to distinguish between different cataract subtypes, including subcortical, nuclear, and posterior subcapsular subtypes, decreases the statistical power to identify cataract-associated alleles. Since most genome-wide studies fail to adjust such covariates, future investigation into the gene–environment interaction is needed.

In conclusion, in our study, we analyzed the data from the TWB2.0 and TWB1.0 databases. A total of 167 and 43 cataract-related SNPs were identified in the older and younger cataract groups, respectively. Further analyses are required to survey the risk loci differences between cataract subtypes. Furthermore, a novel PRS model was built to identify patients susceptible to cataracts in each of the older and younger populations. The model was validated by an independent Han-based cohort from the TWB1.0 database. Overall, the newly identified genome-wide SNP loci, along with the PRS model, highlight the genetic bases of cataracts, open new avenues for molecular research, and present clinical significance for distinguishing high-risk cataract individuals.

## Figures and Tables

**Table 1:** Participant characteristics from TWB 2.0 and TWB 1.0.

	Discovery TWB2.0 (*n* = 20,335)					Validation TWB1.0 (*n* = 7993)					Statistics and *p*-Values (Case < 60) ^2^	Statistics and *p*-Values (Case > 60) ^2^
Variables	Case < 60 (*n* = 1959)	Case ≥ 60 (*n* = 5120)	Control (*n* = 13,256)	*p*-Values (Case < 60) ^1^	*p*-Values (Case ≥ 60) ^1^	Case < 60 (*n* = 757)	Case ≥ 60 (*n* = 2246)	Control (*n* = 4990)	*p*-Values (Case < 60) ^1^	*p*-Values (Case ≥ 60) ^1^		
**Sex**												
Male (%)	490 (25.01)	1500 (29.30)	4898 (36.95)	<2.2 × 10^−16^	<2.2 × 10^−16^	337 (44.12)	998 (44.43)	2610 (52.30)	7.65 × 10^−5^	6.86 × 10^−10^	<2.2 × 10^−16^	<2.2 × 10^−16^
Female (%)	1469 (74.99)	3620 (70.70)	8358 (63.05)			420 (55.48)	1248 (55.57)	2380 (47.70)				
**Age (years)**	54.04 ± 5.34	65.11 ± 3.11	63.68 ± 2.84	<2.2 × 10^−16^	<2.2 × 10^−16^	53.08 ± 6.12	66.24 ± 3.60	64.4 ± 3.37	<2.2 × 10^−16^	<2.2 × 10^−16^	5.079 × 10^−9^	<2.2 × 10^−16^
**BMI**	23.94 ± 3.81	24.13 ± 3.41	24.42 ± 3.42	1.46 × 10^−7^	2.071 × 10^−7^	24.3454 ± 3.713	24.3618 ± 3.330	24.4594 ± 3.231	0.4242	0.2448	0.09964	0.05096
**Diabetes**												
No (%)	1771 (90.40)	4284 (83.67)	12,028 (90.74)	<2.2 × 10^−16^	<2.2 × 10^−16^	650 (85.87)	1862 (82.90)	4457 (89.32)	<2.2 × 10^−16^	8.636 × 10^−7^	0.04622	0.4888
Yes (%)	188 (9.60)	836 (16.33)	1228 (9.26)			107 (14.13)	384 (17.10)	533 (10.68)				
**Hypertension**												
No (%)	1632 (83.31)	3607 (70.45)	9948 (75.05)	<2.2 × 10^−16^	<2.2 × 10^−16^	632 (83.49)	1530 (68.12)	3634 (72.83)	<2.2 × 10^−16^	<2.2 × 10^−16^	0.5577	0.01028
Yes (%)	327 (16.69)	1513 (29.55)	3308 (24.95)			125 (16.51)	716 (31.88)	1356 (27.14)				
**Hyperlipidemia**												
No (%)	1691 (86.32)	4106 (80.20)	11,431 (86.23)	<2.2 × 10^−16^	<2.2 × 10^−16^	653 (86.26)	1806 (80.41)	4260 (85.37)	<2.2 × 10^−16^	<2.2 × 10^−16^	0.8507	0.2707
Yes (%)	268 (13.68)	1014 (19.80)	1825 (13.77)			104 (13.74)	440 (4.85)	730 (14.93)				
**Asthma**												
No (%)	1886 (96.27)	4899 (95.68)	12,827 (96.76)	<2.2 × 10^−16^	3.394 × 10^−16^	721 (95.24)	2137 (95.15)	4838 (96.95)	<2.2 × 10^−16^	0.007776	0.1738	0.03337
Yes (%)	73 (3.73)	221 (4.32)	429 (3.24)			36 (4.76)	109 (4.85)	152 (3.05)				
**GFR**												
>60 (%)	1913 (97.70)	4899 (95.78)	12,828 (96.79)	0.03452	0.0009946	736 (97.23)	2115 (94.17)	4814 (96.51)	0.3662	5.97 × 10^−6^	0.02036	5.6 × 10^−8^
<60 (%)	45 (2.30)	216 (4.22)	426 (3.21)			21 (2.77)	131 (5.83)	174 (3.59)				

^1^ *p*-values for age and BMI were calculated by Student’s *t*-test, whereas the other characteristics were calculated by chi-squared tests. ^2^ *p*-values for comparison between the means of the discovery cohort and the validation cohort. Abbreviations: BMI = body mass index; GFR = glomerular filtration rate.

**Table 2:** Selected cataract-associated SNPs identified by GWAS in TWB2.0 ^1^.

Population	SNP ^1^	CHR	Position	MAF (in Cases)	MAF (in Controls)	*p*-Value	OR	adj. P	Nearest Gene
	rs7513180	1	63874130	0.03579	0.02373	7.39 × 10^−6^	1.527	2.91 × 10^−6^	ROR1
	rs117994780	2	71677869	0.02517	0.01539	8.69 × 10^−6^	1.651	1.29 × 10^−5^	DYSF
	rs237885	3	8753857	0.2696	0.305	7.55 × 10^−6^	0.8412	2.57 × 10^−6^	OXTR
	rs3814411	3	112333058	0.02214	0.01307	8.50 × 10^−6^	1.709	1.98 × 10^−5^	CD200
	rs143616043	5	51456733	0.02783	0.04289	9.11 × 10^−6^	0.6389	7.82 × 10^−6^	ISL1
Younger	rs146654893	9	21619135	0.01959	0.01118	9.11 × 10^−6^	1.766	2.67 × 10^−5^	F2Z2F3
Population	rs117753381	10	10644914	0.02692	0.01619	2.15 × 10^−6^	1.681	3.29 × 10^−6^	CELF2
(<60)	rs77137422	12	20324909	0.04933	0.03411	2.03 × 10^−6^	1.469	1.04 × 10^−5^	PDE3A
	rs9788929	16	16829414	0.1914	0.1625	6.27 × 10^−6^	1.22	3.30 × 10^−5^	XYLT1
	rs374431	19	58279347	0.4243	0.4625	7.44 × 10^−6^	0.8563	1.07 × 10^−5^	ZNF8-ERVK3-1
	rs13046594	21	38436779	0.05021	0.03464	1.51 × 10^−6^	1.473	3.97 × 10^−6^	ERG
	rs738096	22	17773177	0.3911	0.4289	8.24 × 10^−6^	0.8552	1.78 × 10^−5^	BID
	rs76079963	22	48857843	0.03724	0.02495	8.57 × 10^−6^	1.511	1.33 × 10^−4^	TAFA5
	rs140318176	2	125365220	0.01348	0.02041	9.91 × 10^−6^	0.656	3.95 × 10^−5^	-
	rs11133245	4	53154174	0.1834	0.2045	6.53 × 10^−6^	0.8737	7.65 × 10^−6^	SCFD2
	rs145208055	4	8895796	0.02181	0.01371	2.82 × 10^−8^	1.604	4.61 × 10^−7^	HMX1
	rs1521224	6	121973799	0.06622	0.08051	4.15 × 10^−6^	0.81	5.45 × 10^−6^	HSF2
	rs9345070	6	91015542	0.4682	0.4958	2.05 × 10^−6^	0.8952	3.39 × 10^−6^	MAP3K7
Older	rs74774546	6	121787961	0.1254	0.1461	3.32 × 10^−7^	0.8378	1.10 × 10^−6^	GJA1
Population	rs4726966	7	148387557	0.04967	0.06191	7.70 × 10^−6^	0.7919	2.23 × 10^−5^	CNTNAP2
(≥60)	rs148814099	9	89141883	0.01917	0.01285	7.57 × 10^−6^	1.501	2.44 × 10^−5^	SHC3
	rs10781570	10	132372299	0.1387	0.1214	8.37 × 10^−6^	1.166	3.21 × 10^−5^	LRRC27
	rs28503213	18	77663436	0.2459	0.2238	6.25 × 10^−6^	1.131	3.40 × 10^−5^	GALR1
	rs2272537	19	35704684	0.1347	0.1173	6.83 × 10^−6^	1.171	2.36 × 10^−6^	ZBTB32
	rs56792854	19	35737488	0.1316	0.1147	8.82 × 10^−6^	1.17	2.96 × 10^−6^	KMT2B
	rs60128322	19	35768908	0.1351	0.1179	7.63 × 10^−6^	1.169	1.61 × 10^−6^	PROSER3

^1^ All genome-wide significant SNPs for each independent locus were identified in the TWB2.0 Biobank. For a complete list of cataract risk SNPs (*p* < 1 × 10^−4^), please refer to Table S1 (<60 years old), Table S2 (≥60 years old), and Table S3 (all). Abbreviations: SNP = single nucleotide polymorphism; CHR = chromosome; MAF = minor allele frequency; OR = odds ratio.

**Table 3:** Comparison of the predictive performance of PRS with different tuning parameters.

	Case < 60				Case > 60			
Tuning Parameters ^1^	N SNPs	Mean PRS		AUC (95% CI)	Top N SNPs Included	Mean PRS		AUC (95% CI)
		Case	Control	TWB2.0	for PRS Calculation	Case	Control	TWB2.0
*p* ≤ 10^−4^ and r^2^ < 0.2	95	0.0733	0.0130	0.7129 (0.6996, 0.7262)	131	0.0138	−0.0250	0.6693 (0.6597, 0.6790)
*p* ≤ 10^−4^ and r^2^ < 0.04	90	0.0818	0.0238	0.7102 (0.6969, 0.7235)	130	0.0152	−0.0231	0.6697 (0.6600, 0.6793)
*p* ≤ 2.5 × 10^−4^ and r^2^ < 0.2	228	0.1925	0.0366	0.7874 (0.7756, 0.7993)	292	0.0404	−0.0616	0.7383 (0.7295, 0.7472)
*p* ≤ 2.5 × 10^−4^ and r^2^ < 0.04	218	0.2046	0.0555	0.7862 (0.7743, 0.7980)	287	0.0415	−0.0595	0.7385 (0.7296, 0.7473)
*p* ≤ 5 × 10^−4^ and r^2^ < 0.2	428	0.3733	0.0602	0.8528 (0.8430, 0.8626)	547	0.1099	−0.0896	0.7907 (0.7826, 0.7987)
*p* ≤ 5 × 10^−4^ and r^2^ < 0.04	415	0.3814	0.0810	0.8527 (0.8429, 08625)	535	0.1134	−0.0830	0.7903 (0.7822, 0.7983)
*p* ≤ 7.5 × 10^−4^ and r^2^ < 0.2	643	0.5352	0.0641	0.8915 (0.8833, 0.8998)	809	0.2018	−0.0912	0.823 (0.8156, 0.8305)
*p* ≤ 7.5 × 10^−4^ and r^2^ < 0.04	617	0.5358	0.0836	0.8913 (0.8831, 0.8996)	787	0.2065	−0.0810	0.8226 (0.8151, 0.8300)
*p* ≤ 10^−3^ and r^2^ < 0.2	838	0.6788	0.0521	0.9166 (0.9095, 0.9237)	1024	0.2918	−0.0917	0.8464 (0.8394, 0.8533)
*p* ≤ 10^−3^ and r^2^ < 0.04	804	0.6716	0.0700	0.9165 (0.9094, 0.9236)	991	0.2942	−0.0812	0.8461 (0.8391, 0.8530)

^1^ Tuning parameters, including genome-wide significance (*p*) and r^2^ for LD clumping. The table shows that the mean PRS is higher among the cases than the controls across all PRS models. Abbreviations: SNP = single nucleotide polymorphism, PRS = polygenic risk score; AUC (95% C.I) = area under curve (95% confidence interval); TWB2.0 = Taiwan Biobank 2.0.

**Table 4:** Distribution of cataract cases and controls regarding PRS quantiles in younger and older groups.

		(min,Q1)	(Q1,Q2)	(Q2,Q3)	(Q3,Q4)
	Case <60, N = 1567	77	159	382	949
Younger	(age < 60%)	4.91%	10.15%	24.38%	60.56%
Population	Control, N = 10,603	2965	2884	2660	2094
(age < 60) ^1^	(n,%)	27.96%	27.20%	25.09%	19.75%
	**OR for case (95% C.I)**	**1**	**2.12 (1.62, 2.81)**	**5.52 (4.33, 7.15)**	**17.45 (13.84, 22.33)**
	Case >60, N = 4095	341	691	1119	1944
Older	(age ≥ 60,%)	8.33%	16.87%	27.33%	47.47%
Population	Control, N = 10,603	3333	2984	2555	1731
(age ≥ 60) ^1^	(n,%)	31.43%	28.14%	24.10%	16.33%
	**OR for case (95% C.I)**	**1**	**2.26 (1.97, 2.60)**	**4.28 (3.75, 4.89)**	**10.97 (9.66, 12.50)**

^1^ PRS_younger was used to assess the younger population (<60 years old), while PRS_older was used to assess the older population (≥60 years old) Abbreviations: OR = odds ratio with the reference being the lowest PRS quantile group (min,Q1); Q = quantile; 95% C.I. = 95% confidence interval.

**Table 5:** Risk of high PRS groups for development of cataracts for younger cases (<60) and older cases (≥60).

High PRS Group	Reference Group	OR for Case < 60 (95% C.I)	OR^1^ for Case ≥ 60 (95% C.I)
Top 25%	Remaining 75%	6.24 (5.58, 6.98)	4.63 (4.28, 5.02)
Top 20%	Remaining 80%	6.26 (5.59, 7.00)	4.77 (4.38, 5.20)
Top 10%	Remaining 90%	7.09 (6.22, 8.08)	5.48 (4.89, 6.14)
Top 5%	Remaining 95%	9.16 (7.73, 10.85)	6.74 (5.74, 7.94)

Abbreviations: PRS = polygenic risk score model; OR (95% C.I.) = odds ratio (95% confidence interval).

## Data Availability

Publicly available data were downloaded from the following databases: the TWB genetic and phenotype datasets are available through the TWB: https://www.twbiobank.org.tw, accessed on 25 February 2022; BBJ summary statistics: http://jenger.riken.jp/en/result, accessed on 25 February 2022; UKBB summary statistics: https://pheweb.org/UKB-Neale, accessed on 25 February 2022; GWASCatlog summary statistics: https://www.ebi.ac.uk/gwas/summary-statistics, accessed on 25 February 2022.

## Supplementary Materials

The following supporting information can be downloaded at: https://www.mdpi.com/article/10.3390/biomedicines10081920/s1, Table S1: Cataract-associated SNPs identified by GWAS among the young cases (<60 years old), Table S2: Cataract-associated SNPs identified by GWAS among the old cases (≥60 years old), Table S3: Cataract-associated SNPs identified by GWAS (case all).
